# Intellectual Structure of Coronavirus Research: A Perspective From an Author Cocitation Analysis

**DOI:** 10.3389/frma.2020.595370

**Published:** 2020-11-10

**Authors:** Junyi Mei, Dangzhi Zhao, Andreas Strotmann

**Affiliations:** ^1^ The Max Rady College of Medicine, University of Manitoba, Winnipeg, MB, Canada; ^2^ School of Library and Information Studies, University of Alberta, Edmonton, AB, Canada; ^3^ ScienceXplore, Bad Schandau, Germany

**Keywords:** COVID-19, author co-citation analysis, intellectual structure, bibliometrics, coronavirus

## Abstract

The present study examines the intellectual structure of research on coronavirus, as revealed from an author co‐citation analysis using citation data retrieved from the Web of Science Core Collection and mapped to the PubMed database. Four major dimensions are identified: I) outbreaks, II) viral structure and function, III) vaccine and therapeutic development, and IV) coronaviruses found in a range of animals. The “outbreaks” dimension is by far the most prominent, dominated by reports on the three recent major outbreaks: COVID-19, severe acute respiratory syndrome, and Middle East respiratory syndrome. The focus of research on major outbreaks is on public health and clinical research, with focus on disease characterization, diagnosis, transmission, and clinical course. Notably, certain clinically important areas, such as mental health during outbreaks and viral surveillance, among others, did not stand out as identifiable specialties or topics in the coronavirus research landscape. Results from this study should contribute to the understanding of the coronavirus research landscape and to the identification of strengths and weaknesses of current research on COVID-19.

## Introduction

According to public health data compiled by Johns Hopkins University (https://coronavirus.jhu.edu/map.html), as of August 14, 2020, just 5 months after the WHO declared the new coronavirus (COVID-19) a pandemic, more than 21 million cases of COVID-19 and 761,000 related deaths have been reported in 188 countries and territories. The pandemic has disrupted social and economic systems worldwide, triggering a deep global recession (Gopinath, 2020; Nicola et al., 2020; The New York Times Editorial, 2020).

While citizens, organizations, and governments have been working together to contain the virus, doctors and scientists have been working around the clock to study the virus and the pandemic. They have been trying to understand the novel virus and the impact of the pandemic on people’s mental and physical health and to find effective treatments and preventive measures. Their efforts have drawn on knowledge and experiences accumulated from studying and fighting coronaviruses and outbreaks in the past, including the 2002 severe acute respiratory syndrome (SARS) and the 2012 Middle East respiratory syndrome (MERS) outbreaks.

The present study examines the intellectual structure of research on coronavirus in all years as revealed from an author cocitation analysis (ACA). Results could contribute to the understanding of the coronavirus research landscape and to the identification of strengths and weaknesses of current research on COVID-19.

## Materials and Methods

### Author Cocitation Analysis

ACA has been used successfully to study the intellectual structures of the information science field and a number of other research fields (Persson, 1994; White and Griffith, 1981; White and McCain, 1998; Zhao and Strotmann, 2008a; Zhao and Strotmann, 2011a; Zhao and Strotmann, 2014). ACA is one of the major bibliometric methods for studying intellectual structures of research fields and has been frequently examined and improved upon since it was first introduced (White and Griffith, 1981; Zhao and Strotmann, 2008a). Compared to document- or journal-based cocitation analysis methods, ACA uses “author” as the unit of analysis, which has “the advantage of a nice balance in granularity and the potential for studying people in addition to their works (White, 1990)” (Zhao and Strotmann, 2014, p. 996).

ACA uses the overlap of citations to two authors’ oeuvres (i.e., their cocitation count) to measure how closely these two oeuvres are related in terms of subject matters or methodological approaches, where an author’s oeuvre is defined as the full set of articles that an author has written (McCain, 1990) or in practice that part of it which has been indexed in the citation databases used for data collection. Highly cited authors in a research field are usually selected to represent the field being studied, and their matrices of cocitation counts can then be analyzed using multivariate statistical analysis methods and visualized *via* network analysis tools. Author clusters can be interpreted as specialties and their positions in the network as interrelationships between researchers and specialties.

### Data Collection

We downloaded all 25,137 full records retrieved on June 28, 2020, from the following search string in the “Topic” fields in the Web of Science (WoS) Core Collection for all years:

“COVID-19” OR Coronavirus OR “Corona virus” OR “2019-nCoV” OR “SARS-CoV” OR “MERS-CoV” OR “Severe Acute Respiratory Syndrome” OR “Middle East Respiratory Syndrome”.

From these records, we removed those that did not contain cited references or did not have PubMed IDs and matched the remaining 23,682 (or 94%) WoS records to corresponding PubMed records using their PubMed IDs. We then matched the vast majority (358,662) of the cited references in these WoS records to their corresponding full PubMed records using their DOIs, which resulted in a total of 85,924 distinct PubMed records. Additional matching strategies as described in Strotmann and Zhao (2010) did not produce significantly better results, presumably because the coronavirus literature as indexed in WoS references a significant amount of nonbiomedical literature that is simply not indexed in PubMed. These PubMed records and the citation links between them recorded from the downloaded WoS records constitute our dataset for this study.

Reasons for the additional step of matching WoS records to PubMed records and then using the latter as a dataset were discussed in Strotmann and Zhao (2010, 2015). In a nutshell, compared to WoS records, PubMed records are professionally indexed with high-quality metadata and marked up in XML, which improves the accuracy of data for processing. They are particularly useful in effective automatic author name disambiguation.

### Author Name Disambiguation

Alarming results have been reported from research on the effects of name ambiguities on the results of popular types of social or bibliometric network analysis: both typical evaluative citation analyses of individuals and some of the most basic statistical features of realistic large-scale networks are affected significantly by such ambiguities (Strotmann and Zhao, 2012; Diesner and Carley, 2013; Fegley and Torvik, 2013; Zhao and Strotmann, 2015).

Name ambiguities in citation data sources are significant in many research fields in science and technology partly because of the rising contribution of Asian countries such as China, South Korea, and Vietnam where personal names written in romanized form and indexed as last name plus first name initial are highly ambiguous (Strotmann and Zhao, 2012). Author name disambiguation is therefore essential for any ACA of research on coronavirus because of the large number of Chinese authors involved. Since both the 2002 SARS and the COVID-19 outbreak started in China, Chinese doctors and scientists had first-hand data ahead of the rest of the world and therefore were among the first and most active in research on COVID-19. Contrary to names in many other cultures, Chinese and Korean names are featured with a small number of last names and a vast variety of first names that are often unique when written in Chinese characters but identical when written according to a romanization scheme such as Pinyin or Wade-Giles.

For the present paper, we therefore used a slightly updated version of the author name disambiguation algorithm described in Strotmann et al. (2009), which primarily relies on the structure of the full coauthor network of all citing and cited authors for attribution of cited references to author oeuvres. The algorithm identified a total of 508,862 individual authors in our dataset; 63,754 of whom as first authors of publications.

### Data Analysis

#### Citation and Cocitation Counting

We processed our dataset after author name disambiguation to identify the 300 authors whose first-author oeuvres were most highly cited in our dataset to represent the field of research on coronavirus.

For these 300 authors, we calculated their cocitation count matrix using first-author-only counting. As in previous studies, the cocitation count of authors A and B is the total number of articles that cite at least one document from A’s oeuvre and at least an additional one from B’s where an author’s oeuvre is the complete set of documents written by that author as the first author (McCain, 1990). The cocitation count of authors A and B is thus defined mathematically as the size of the intersection of the two sets of documents that cite A’s and B’s oeuvres, respectively. We calculated meaningful diagonal values for the diagonal of the cocitation matrix, i.e., we counted only those citing papers that included at least two references to A’s oeuvre in the count for the cocitation count of A’s oeuvre with itself, thus assuring that all cocitation counts are based on two distinct cited references in the citing paper (Zhao and Strotmann, 2008b).

We chose first-author rather than all-author citation and cocitation counting here, an unusual choice for a study of a biomedical research field where collaborative research is the norm. This choice was made to support our aim for a more detailed view of the overall structure of the coronavirus research landscape given the complexity of the topic area that was to be expected. The following findings from Zhao and Strotmann (2011b), which studied the highly collaborative stem cell research based on a comprehensive, high-quality dataset subjected to automatic reference and author name disambiguation, explain our reasoning for this unusual choice:

- “All three types of ACA, i.e., first-author, last-author, and fractional all-author, produce surprisingly similar results in terms of the overall structure of the stem cell field that they reveal given the highly collaborative nature of the field, but they also differ with respect to the degree of detail of major areas of studies shown and with respect to the specific set of specialties identified.” (p. 673)
- “First-author counting tends to identify researchers who have conducted highly influential studies and emphasize a researcher’s unique areas of study and most influential contributions. First-author ACA, therefore, shows a considerably more detailed picture with more fragmentation within the major clusters of specialties of the field than all-author ACA does.” (p. 674)

We also used a much larger number of highly cited authors to represent the research field compared to previous ACA studies (300 vs. 120, e.g.) in order to improve chances for representative authors of some of the less prominent subfields to show up in our analysis results.

#### Factor Analysis and Visualization

A factor analysis of the cocitation matrix of the 300 highly cited authors was conducted using the Python factor-analyzer package (https://pypi.org/project/factor-analyzer/) to explore the underlying structure of the interrelationships between the selected authors. Factors were extracted by Principal Component Analysis, and the number of factors extracted was determined using Kaiser’s rule of eigenvalue greater than one. This resulted in a 29-factor model that explains 84.3% of the total variance.

We applied an oblique rotation to this factor model in the factor analysis, resulting in a pattern matrix and a structure matrix. We visualized the pattern matrix where a loading represents an author’s unique contribution to a factor. To this end, the pattern matrix is “converted one-to-one into a bipartite graph format. This graph is loaded into Pajek for Kamada-Kawai automatic layout using author loadings on factors as similarity measures” (Zhao and Strotmann, 2011b, p. 661).

In the visualization, authors are represented by square nodes and factors by circular nodes, with factors and their members being color-coded. The size of an author node corresponds to the total citation count of the author, and the size of a factor node is determined by the sum of the citation counts of all authors who load sufficiently on its factor (i.e., with a value of 0.3 or higher in the pattern matrix in this case), weighted by their loadings on the factor. For example, if an author has a citation count of 100 and loads 0.8 on a factor, this author adds 100 × 0.8 = 80 to the sum representing this factor’s approximate total citation count. The weighting attempts to take into account that an author may contribute to several specialties, but only the part of the author’s oeuvre that corresponds to this specialty should be counted. Node sizes show the relative prominence of authors and specialties measured by citation counts, an obviously important feature of the intellectual structure of a research field.

#### Interpretation of Results

We interpreted large factors as specialties and small factors as topics. What specialty or topic each factor represents is determined by looking for common themes from articles written by authors who load primarily in each factor through a close reading of titles and sometimes abstracts of these articles. A factor is labeled as undefined (UD) if all loadings in this factor are lower than 0.7, although an attempt may still be made at interpreting it.

We use the highest loading of a factor to indicate its distinctiveness. The size of a factor is defined as the number of authors who load primarily on this factor in the pattern matrix. The size of a factor node (circle) on the maps, however, is the weighted sum of the citation counts of all authors in this factor as discussed above. Both sizes indicate the relative prominence of a specialty in the research field, one by the number of authors working on the specialty and the other by these authors’ collective citation impact.

### Limitations

We limited our dataset to publications and their cited references that were indexed in PubMed, which captures almost all international biomedical research on coronavirus but may have largely excluded social science studies as they tend to be absent in PubMed.

“Although ACA has long been shown to be an effective method for eliciting a bird’s-eye view of the intellectual structure of a research field, there are some limitations to ACA, as with any methodology” (Zhao and Strotmann, 2011b, p. 673). One limitation is that it is less effective in detecting research fronts. ACA normally relies on highly cited authors to represent the research field whose intellectual structure is being studied. What ACA reveals is therefore the structure of the knowledge base rather than the research fronts of research fields. This limitation is especially noticeable in fast-moving research fields such as current research on coronavirus. Other methods such as author bibliographic coupling analysis are more effective in detecting research fronts (Zhao and Strotmann, 2008a; Zhao and Strotmann, 2014).

## Results

Four major dimensions can be identified from the data as explained below: (I) outbreaks, (II) viral structure and function, (III) vaccine and therapeutic development, and (IV) coronavirus in various animals.

Table 1 presents the specialties and the major dimensions they belong to. Their distinctiveness and prominence (= size) are indicated, respectively, by the highest loading and the number of authors among the 300 most highly cited who load primarily on each factor.

**TABLE 1:** Overview of a 29-factor model.

Factor number	Label	Dimension	Size	Highest loading
F1	COVID-19 outbreak 2019/2020	I	57	1.06
F2	SARS outbreak 2002	I	31	1.07
F3	MERS outbreak 2012	I	28	1.08
F4	RNA transcription	II	15	1.12
F5	Replication	II	11	1.33
F6	Outbreaks of other human coronaviruses	I	14	1.17
F7	Characterization of viruses in bats	I	12	1.11
F8	Internalization of murine coronaviruses	II	13	1.06
F9	SARS vaccine development	III	13	1.03
F10	Gene expression/translation	II	12	1.1
F11	Release	II	12	1.15
F12	Feline coronaviruses	IV	8	1.01
F13	Internalization	II	8	1.08
F14	Porcine delta coronavirus and epidemic diarrhea virus	IV	7	1.05
F15	3CL protease as target	III	6	1.07
F16	Animal models	III	6	0.75
F17	MERS spike protein as target	III	2	0.78
F18	Avian	IV	6	1.04
F19	Porcine TGEV and PRCV	IV	7	1.03
F20	Early findings about coronavirus	II	5	0.75
F21	SARS nucleocapsid protein	II	5	0.98
F22	UD (clinical intensive care)	I	0	—
F23	ACE2	III	4	0.75
F24	Bovine coronaviruses	IV	5	0.94
F25	Canine coronaviruses	IV	4	1.05
F26	Model forecasts of epidemics	I	1	0.8
F27	UD (clinical treatment regimes)	I	2	0.66
F28	UD (papain-like protease)	II	3	0.63
F29	CNS involvement of murine coronavirus	II	2	1.01

**FIGURE 1:**
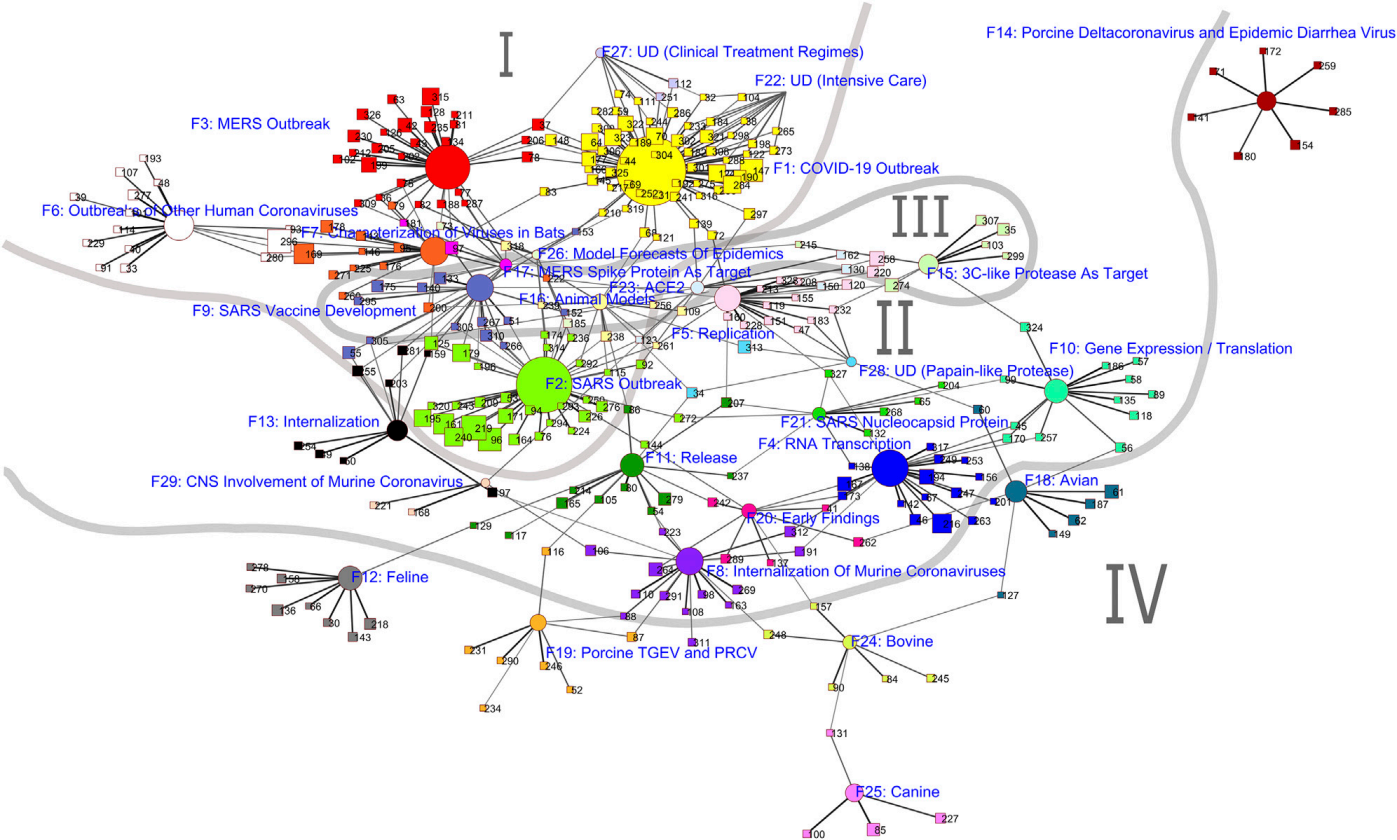
Visualization of the intellectual structure of research on coronavirus.

The four major dimensions identified are also marked with their numbers (I–IV) and with boundary lines between them in Figure 1 which visualizes the intellectual structure of coronavirus research identified using the methods described above. Circular notes are factors marked with their labels and factor numbers which correspond to labels and numbers in Table 1. Square nodes represent authors. Due to the large number of authors in the network, we chose to label author nodes with their numbers instead of their names in order to reduce the crowdedness and increase the readability of the map. Author names corresponding to these numbers are provided in Table 2 (for the top 30 highly cited authors) and as Supplementary Materials (for all authors) along with their citation counts and the specialties they belong to. Node size on the map reflects the authors’ individual or a factor’s members' collective (i.e., a specialty’s) citation impact.

**TABLE 2:** Top 30 highly cited authors examined with author cocitation analysis.

Citation rank	Author name	Times cited as first author	Node number	Specialty
1	Peiris, Joseph S. Malik	1,936	219	F2: SARS
2	Woo, Patrick Chiu-Yat	1,757	296	F6: other outbreaks and F7
3	Drosten, Christian	1,700	96	F2: SARS
4	Huang, Chao-Lin	1,629	147	F1: COVID-19
5	Ksiazek, Thomas G.	1,330	161	F2: SARS
6	Lau, Susanna K. P.	1,265	169	F7: viruses in bats
7	Page, G. S.	1,170	216	F4: RNA transcription
8	Rota, Paul A.	1,086	240	F2: SARS
9	Li, Wenhui	1,008	179	F2: SARS and F9 vaccine
10	Wang, Da-Wei	994	284	F1: COVID-19
11	Guan, Wei-Jie	948	124	F1: COVID-19
12	Zaki, Ali M.	931	315	F3: MERS
13	Marra, Marco A.	918	195	F2: SARS
14	Chen, Nanshan	918	70	F1: COVID-19
15	Chan, Jasper Fuk-Woo	903	64	F1: COVID-19
16	Zhu, Na	891	323	F1: COVID-19
17	Zhou, Peng	760	322	F1: COVID-19
18	Memish, Ziad A.	728	199	F3: MERS
19	Lee, Nelson	714	171	F2: SARS
20	Makino, Shinji	692	194	F4: RNA transcription
21	Snijder, Eric J.	656	258	F5: replication
22	Li, Qun	640	177	F1: COVID-19
23	Assiri, Abdullah	623	42	F3: MERS
24	Lai, M. M.	606	167	F4: RNA transcription
25	Cavanagh, D.	599	61	F18: avian
26	Guan, Yi	580	125	F2: SARS
27	Sturman, L. S.	577	264	F8: internalization
28	Zhou, Fei	558	321	F1: COVID-19
29	Thiel, Volker	556	274	F15: 3CL protease as target
30	Du, Lanying	513	97	F17: spike protein as target

### Dimension I: Outbreaks

Studies on the three major outbreaks in recent years are the most active research areas, which form a triangle on the top left part of Figure 1. The focus is largely on topics relevant to public health and clinical research, such as disease characterization, diagnosis, transmission, and clinical course. The specialty representing studies of the current COVID-19 outbreak (F1) is by far the largest and is almost double the size of those of the SARS and MERS outbreaks in 2003 and 2012, respectively (F2 and F3). This reflects the actual scale and severity of this ongoing global pandemic compared to the other two outbreaks that were more or less localized within a single region (i.e., Asia and the Middle East). The rapidly spreading virus has made studying, understanding, and containing it, as well as treating the resulting disease an urgent agenda item worldwide. New technologies have also contributed to swift research and development. For example, 2 months after the outbreak was first identified in December 2019 in China, “scientists in China [had] sequenced the genome of the COVID-19 virus demonstrating that it is a completely new virus, albeit closely related to the coronavirus (CoV) responsible for SARS” (Asian Scientist Magazine, 2020). Eight months after that, seven vaccines have already reached phase 3 clinical trial, which is “a scientific quest moving at record-breaking speed” (Steckelberg et al., 2020). Furthermore, the accuracy and speed of viral tests for COVID-19 have been increasing, and the results can now be available in minutes (FDA, 2020).

The COVID-19 outbreak specialty is weakly (through four authors) connected to the MERS outbreak specialty but had only indirect connections with the SARS outbreak specialty *via* the specialties on models for SARS infection and clinical course (F16) or for forecasting epidemics (F26). The SARS and MERS outbreak specialties are strongly connected *via* specialties related to vaccine development (F9, F17) or to the characterization of viruses in bats (F7).

The weakness of direct links between the separate outbreaks is likely due to not only their separation in time and geographic areas affected but also the segregation of the medical literature of these separate regions. From a public health perspective, the priority is to characterize the current outbreak and its distinct source, transmission characteristics, and clinical course so that countermeasures can be rapidly put in place. Often an outbreak is first established as reflected by a number of case reports published in medical journals, which is seen in the COVID19 outbreak specialty and the two subtopics that stand out from it, intensive care and treatment regimes. As outbreaks are usually time-limited events, there is a surge and decline in the number of articles published. The focus then shifts to surveillance, coronavirus vaccine development, and prevention and control of similar outbreaks in the future. Following the lead of the SARS and MERS outbreaks, the model will likely shift as more articles on COVID-19 vaccine development and outbreak prevention and control emerge in the coming months to years.

Although bats are believed to be the origin of the coronaviruses that caused all three major outbreaks, unlike the strong link between the SARS and MERS outbreaks with the characterization of viruses in bats specialty, only a single author (Zhao, Jin-Cun) has been perceived to be relevant to both the characterization of viruses in bats specialty and the COVID-19 outbreak specialty. Although there might be political factors in play limiting research in this area, as a few believe that the COVID-19 virus had been engineered in the laboratory, we expect this link to strengthen as the focus of research efforts shift to outbreak prevention.

Even though seven COVID-19 vaccines have reached phase 3 clinical trials at this time, only three authors link the COVID-19 outbreak specialty with the vaccine and therapeutic development specialties. This seemingly weak connection may have to do with the scientific division where vaccine and therapeutic development is often led and driven by industries whereas public health issues during outbreaks are studied in the public sectors by scientists and doctors (Callaway, 2020).

There is a smaller specialty on outbreaks of a variety of human coronaviruses (e.g., HcoV-NL63, HKU1, OC43, and 229E) in individual countries (F6). The small size is expected as these outbreaks are of a smaller scale and pose less of a global threat. Its focus is also on public health issues. This specialty is only connected to the characterization of viruses in bats specialty (F7), but that connection is strong.

### Dimension II: Viral Structure and Function

A coronavirus particle is composed of an RNA core and four major structural proteins, three of which make up the viral envelope. Coronaviruses are positive-strand RNA viruses. Once they bind to cellular receptors and enter the host cell, their genomic RNA is translated by cellular machinery to produce viral proteins essential to viral replication. Viral replication involves RNA transcription and protein translation and processing. New viral particles are then assembled and released from the host cell and may go on to infect more host cells (Payne, 2017).

Studies on coronavirus structure are intimately related to and required for understanding viral function and infectivity, which then inform vaccine development. Figure 1 reflects these interrelationships.

To the lower right side of the outbreaks triangle on the map is a group of loosely connected specialties on viral structure and function: SARS nucleocapsid protein (F21), murine coronavirus binding (F8), internalization (F13), RNA transcription (F4), gene expression/translation (F10), replication (F5), and release (F11). This group of specialties is connected to the group of specialties on vaccine and therapeutics development mostly *via* internalization (F13) and 3CL protease (F15), which will be further discussed in the next section.

### Dimension III: Vaccine and Therapeutics Development

Viral vaccine development requires understanding of viral structure and function. Different vaccines such as attenuated or inactivated virus, viral vector, nucleic acid, and protein-based vaccines induce the immune response and formation of memory lymphocytes, which impart long-term immunity (Callaway, 2020; Le Bert et al., 2020).

The group of specialties on vaccine and therapeutics development (F9, F15, F16, F17, and F23) is located primarily within the triangle of the three major outbreaks, which is expected since research on vaccine and therapeutics development often begins during and outlives outbreaks as we had mentioned previously. The largest specialty is SARS vaccine development (F9), which Figure 1 shows to have strong links with the internalization specialty (F13) in the viral structure and function dimension. Both the spike protein (F17) and ACE2 (F23) are required for viral internalization, the former on the virus and the latter on the host cell. Spike protein is the main viral envelope protein involved in binding and entering the host cell and is the focus of developing immunogenic epitopes in several vaccines. Both SARS-CoV and SARS-CoV-2 viruses, implicated in SARS and COVID-19 outbreaks, respectively, bind to host ACE2 to enter the host cell, making it a suitable therapeutic target. 3CL protease (F15) is another key therapeutic target as it plays a crucial role in the production of many viral proteins involved in viral replication.

It is interesting to note that other aspects of studies on viral structure and function such as F21, F4, F10, F5, and F11 are only indirectly linked to vaccine and therapeutic development. Most vaccine development studies focus on immunogenic epitopes that are related to viral binding and internalization and less on subsequent viral processes, which reflects differing goals of basic science research compared to translational research. Different researchers may also be involved in these two areas, as the majority of vaccine development is led by industrial and private firms (Callaway, 2020).

### Dimension IV: Coronavirus in Various Animals

On the outermost periphery, we find studies on coronaviruses in various animals beyond bats, the putative origin of the viruses for the three major outbreaks. This group of specialties includes F12 feline, F14 and F19 porcine, F18 avian, F24 bovine, and F25 canine coronaviruses. To note, the specialty on porcine coronavirus (F14) is isolated on the top right corner of the map (with the chosen threshold of loadings greater than 0.3).

These coronaviruses do not appear to be related to COVID-19 or the other two major outbreaks in humans, which is reflected on the visualization (Figure 1) by their extremely weak connection to the rest of the map. Neither are they connected to vaccine development, indicating that the possibility of any of these viruses acting as a natural vaccine the way that the first known vaccine, cowpox, did has not been a primary consideration for coronaviruses. This may change eventually, as Le Bert et al. (2020) report evidence that being infected with animal coronaviruses may in fact confer T-cell immunity to SARS and COVID-19 in humans.

### Top 30 Highly Cited Authors

Table 2 shows that these top 30 authors belong primarily to the SARS and COVID-19 outbreaks, but all four dimensions are represented by these authors which represent only 10% of all the authors included in the ACA. Outside of these two major outbreaks, the MERS outbreak and the RNA transcription specialties are best represented by these highly cited authors, each by three authors. Two additional specialties in the outbreaks dimension (five in total), two additional ones in the viral structure and function dimension (three in total), two in the vaccine and therapeutics development dimension, and one in the coronaviruses in animals dimension are represented, each by a single author.

The top-cited authors within the COVID-19 outbreak specialty appear to be exclusively Chinese researchers and those belonging to the MERS outbreak specialty exclusively researchers from the Middle East countries, which is to be expected given where the respective outbreaks started. It appears that early reports of infection cases and clinical courses have been heavily relied upon in coronavirus research. It is interesting to see that, by contrast, top-cited authors belonging to the SARS outbreak specialty are dominated by researchers from parts of the world other than Asia where the outbreak started and spread primarily. We looked into the case of Christian Drosten (with a citation rank of 3) and found how this German expert on coronavirus gained early access (in March 2003) to a patient with SARS from whom he and colleagues identified the SARS coronavirus. The index patient for Drosten’s highly cited paper that reported this discovery was a physician from Singapore who was “transferred to an isolation unit at the Frankfurt University Hospital with suspected SARS” during a stopover in Frankfurt, Germany, on his flight back to Singapore (Drosten, et al., 2003, p. 1968).

## Discussion

Four major dimensions of coronavirus research have been identified from an ACA of publications reporting research on coronavirus in all years: (I) outbreaks, (II) viral structure and function, (III) vaccine and therapeutics development, and (IV) coronaviruses in various animals. The “outbreaks” dimension is by far the most prominent, dominated by specialties on the three recent major outbreaks: COVID-19 (F1), SARS (F2), and MERS (F3). The focus of research on major outbreaks is on diagnosis, transmission, and clinical course, usually of specific index cases; in the case of COVID-19, clinical intensive care also stands out as a topic. The “outbreaks” dimension and the “vaccine and therapeutics development” dimension are interconnected heavily, as is to be expected. They are loosely connected to the “viral structure and function” dimension which loosely connects to the “coronaviruses in various animals” dimension.

Perhaps just as interesting is what did not appear on this map.

While some aspects of public health, such as epidemiological methods, do appear in the map (F26), research on mental health issues or on the social and economic disruption caused by major outbreaks, especially by the ongoing COVID-19 global pandemic, is missing in the landscape. Missing research on social and economic disruption on the map is expected because such studies are not likely to be indexed in PubMed from which the dataset for the present study was built. Lack of research on mental health issues related to a pandemic may be explained by the added difficulty in recruiting participants for research and in meeting the ethics requirements during a pandemic (Townsend et al., 2020).

Additionally, research on the natural immune response to these viruses in humans, in particular T-cell immunity, or research on how the viruses cause deadly “cytokine storms” and how to prevent or at least ameliorate them did not emerge as stand-alone specialties or identifiable topics in the coronavirus research landscape revealed here. One might have also expected to see research on treatment of COVID-19 with traditional Chinese medicine as an identifiable topic in the landscape, give the controversy around this topic (Cyranoski, 2020; Ni, et al., 2020; Xiong, et al., 2020).

The coronaviruses’ culprit in the three major outbreaks shares many similarities, which raises the question of whether the current outbreak could have been prevented. The answer is likely complicated. Funding, or lack thereof, may have played a role. The SARS and MERS outbreaks were relatively quickly contained, and it appears that vaccine efforts begun during these outbreaks dwindled during the interepidemic period as attention, and funding, shifted elsewhere. Mounting evidence of the potential of coronavirus spillover from bats to humans was not enough to reignite vaccine efforts. A SARS-like virus in bat colonies discovered in Wuhan in 2013 was shown to bind to ACE2 in human cells. Several other strains with similar potential had been discovered. Interspecies spillover of coronavirus from bats to pigs had caused a deadly outbreak in 2016. Nonetheless, the federally funded predict program, with the aim of detecting new pandemic viruses in wildlife across the world, had been terminated just a few months before COVID-19 begun (Schmidt, 2020). On the flip side, it is often said that hindsight is 20/20. Most human coronaviruses cause only mild disease such as the common cold. Governments have a limited amount of funding for vaccine development and may have had other priorities. Political factors may also impair international collaboration, especially when there is the potential for biological weaponry.

Interestingly, research during outbreaks appears to be rapid and abundant and also draws on research conducted during both past outbreaks and interepidemic periods. The process of obtaining ethics approval for studies is slow and can pose a significant barrier to a timely research response. Vaccine development is also notoriously time-consuming, as reflected by the weak connection between COVID-19 and vaccine and therapeutics development 4 months into the pandemic. Outbreaks may also resolve before trials are completed, leading to the publication of incomplete data and inconclusive results. To speed up research response, “regional networks such as PREPARE, ZIKAlliance, ZIKAPlan, ZIKAction, REDe, ISARIC, APPRISE, PANDORA-ID, and ALERRT… with either global or regional reach… run studies or help set up studies in the interepidemic period to bolster preparedness” (London School of Hygiene and Tropical Medicine, 2019). Additionally, to optimize global research response, the WHO has published recommendations for a “‘core protocol,’ which would allow a single clinical trial to extend across multiple infectious disease outbreaks,” and has suggested that “data from a trial that has not yet been completed due to insufficient enrollment should not be released” (Ingeno, 2020). It remains to be seen the results of these efforts and how research during outbreaks evolve in the future.

It will be interesting to revisit this field at later points in time to see if (or when) any breakthroughs in the handling of this class of viruses in medicine and public health appear. At the point of this study, the intellectual landscape we observe is too closely concentrated on the needs of the moment: no such breakthrough appears to have been in sight.

## Data Availability Statement

The data analyzed in this study is subject to the following licenses/restrictions: Data used was downloaded from commercial databases. Subscription is required to access the data. Requests to access these datasets should be directed to https://clarivate.com/webofsciencegroup/solutions/web-of-science/.

## Author Contributions

JM: interpretation of results; writing editing. DZ: study design; data collection; writing; editing. AS; study design; data processing and visualization; editing.

## Conflict of Interest

Author AS is employed by ScienceXplore.

The remaining authors declare that the research was conducted in the absence of any commercial or financial relationships that could be construed as a potential conflict of interest.
